# Suitability of a Non-Dispersive Infrared Methane Sensor Package for Flux Quantification Using an Unmanned Aerial Vehicle

**DOI:** 10.3390/s19214705

**Published:** 2019-10-29

**Authors:** Adil Shah, Joseph Pitt, Khristopher Kabbabe, Grant Allen

**Affiliations:** 1 Centre for Atmospheric Science, The University of Manchester, Oxford Road, Manchester M13 9PL, UK; joseph.pitt@manchester.ac.uk (J.P.); grant.allen@manchester.ac.uk (G.A.); 2 School of Mechanical, Aerospace and Civil Engineering, The University of Manchester, Oxford Road, Manchester M13 9PL, UK; khristopher.kabbabe@manchester.ac.uk

**Keywords:** methane, non-dispersive infrared, lightweight sensor, unmanned aerial vehicle, flux

## Abstract

Point-source methane emission flux quantification is required to help constrain the global methane budget. Facility-scale fluxes can be derived using in situ methane mole fraction sampling, near-to-source, which may be acquired from an unmanned aerial vehicle (UAV) platform. We test a new non-dispersive infrared methane sensor by mounting it onto a small UAV, which flew downwind of a controlled methane release. Nine UAV flight surveys were conducted on a downwind vertical sampling plane, perpendicular to mean wind direction. The sensor was first packaged in an enclosure prior to sampling which contained a pump and a recording computer, with a total mass of 1.0 kg. The packaged sensor was then characterised to derive a gain factor of 0.92 ± 0.07, independent of water mole fraction, and an Allan deviation precision (at 1 Hz) of ±1.16 ppm. This poor instrumental precision and possible short-term drifts made it non-trivial to define a background mole fraction during UAV surveys, which may be important where any measured signal is small compared to sources of instrumental uncertainty and drift. This rendered the sensor incapable of deriving a meaningful flux from UAV sampling for emissions of the order of 1 g s^−1^. Nevertheless, the sensor may indeed be useful when sampling mole fraction enhancements of the order of at least 10 ppm (an order of magnitude above the 1 Hz Allan deviation), either from stationary ground-based sampling (in baseline studies) or from mobile sampling downwind of sources with greater source flux than those observed in this study. While many methods utilising low-cost sensors to determine methane flux are being developed, this study highlights the importance of adequately characterising and testing all new sensors before they are used in scientific research.

## 1. Introduction

The global methane budget is poorly constrained [1], due in part to the lack of accurate quantification of emissions from anthropogenic facility-scale sources [2], such as landfill sites [3,4], oil and gas infrastructure facilities [5,6] or herds of cattle [7,8]. With average annualised atmospheric global methane mole fraction ([*X*]) on the increase [9,10], it is essential that emission fluxes from facility-scale point sources are accurately quantified, using top-down (atmospheric measurement-based) methods, in order to validate bottom-up (component-based) flux estimates [11,12].

A range of flux quantification techniques can be used to derive facility-scale top-down flux estimates using either remote sensing or in situ sampling [13,14]. Remote sensing provides a greater spatial sampling extent at the expense of reduced spatial resolution [15] by either facing towards the emission plume [16,17] or by sampling downwards from above it [18,19,20]. For example, partial column mole fractions can be derived from satellites [21], but natural phenomena such as cloud cover and scene heterogeneity can cause retrieval issues [22]. Near-infrared satellites can only be used in daytime and over land [15]. Most remote sensing techniques can only provide column-integrated (or partial column) measurements [23]. In situ techniques allow sampling to take place from within the emission plume itself [24,25] but as a consequence, spatial sampling coverage is limited to the spatial extent of the sampling platform [26]. In situ [*X*] measurements on a vertical downwind flux plane, near to source, can be used to derive a flux by employing a variety of techniques [14] such as mass balance box modelling [27,28], the tracer dispersion method [29,30] or a Gaussian plume inversion [31,32]. The near-field Gaussian plume inversion (NGI) method, described by Shah et al. [33], is an example of a traditional flux quantification technique, adapted for near-field sampling.

An unmanned aerial vehicle (UAV) is an ideal platform from which to acquire near-field in situ [*X*] measurements [20]. UAVs can probe the planetary boundary layer down to ground level [34], are versatile and are far cheaper than large research aircraft [35]. Furthermore, small lightweight UAV (SLUAV) platforms have fewer airspace restrictions in many jurisdictions, compared to UAVs of over 20 kg (in the UK for example) [36]. A number of strategies can be employed to acquire in situ [*X*] measurements from a SLUAV. Air samples can be captured on board a SLUAV for subsequent analysis [37,38]. However, this results in a poor sampling frequency. Alternatively, air can be pumped through a long tube from a SLUAV to an in situ sensor on the ground [39,40]. However, this can result in issues such as enhanced lag time through the tube and mixing within the tube. In situ [*X*] measurements may also be obtained from a sensor mounted on-board a SLUAV [41]. However this requires a light enough sensor with a sufficient precision, accuracy and resolution for flux quantification, depending on the nature of the source [42,43].

A number of previous studies have featured methane sensors mounted on-board UAVs. For example, Berman et al. [34] mounted a closed path in situ off-axis integrated cavity output spectroscopy sensor (with a 1 Hz precision of ±2 ppb) onto a UAV, but as the sensor had a mass of 19.5 kg, a 200 kg UAV was used to carry the heavy payload. Many in situ wavelength modulation spectroscopy (WMS) sensors (which use tuneable diode lasers) have been mounted onto SLUAV platforms. For example, Liu et al. [44] designed and tested a 3.5 kg closed path WMS sensor on a UAV with a 1 Hz precision of ±79 ppb. Open path WMS sensors have also been used, for example Khan et al. [45] designed and tested a 2 kg open path WMS sensor with a 1 Hz precision of ±2 ppb, which required 2 W of power. Nathan et al. [41] tested a 3.1 kg open path WMS sensor with a 1 Hz precision of 100 ppb, which required 25 W of power. Golston et al. [46] designed and tested a 1.6 kg (including battery) open path WMS sensor with a 1 Hz precision of ±10 ppb, which required 30 W of power. Lightweight sensors are desired for UAV sampling as this maximises flight duration on a given UAV platform.

Non-dispersive infrared (NDIR) spectroscopy uses direct absorption from broad-band infrared radiation, filtered down to a narrow wavelength, to derive mole fraction measurements of a specific gas, depending on wavelength of the filter [23]. Due to its spectroscopic simplicity, NDIR sensors may be small and light enough to mount onto a SLUAV. In this work, the High Performance Platform (HPP) NDIR methane sensor, manufactured by SenseAir AB, was therefore tested to assess its suitability for UAV-derived flux quantification. It was first packaged in an enclosure which was mounted onto a UAV platform. It was then calibrated at a range of water vapour mole fractions, to quantify the variability in the instrumental gain factor. It was then operated on-board a SLUAV, downwind of the controlled release of methane gas, on a vertical flux plane roughly perpendicular to mean wind direction, to test its suitability in flux quantification.

## 2. Materials and Methods

### 2.1. Sensor Packaging and Mounting on an Unmanned Aerial Vehicle

**Figure 1:**
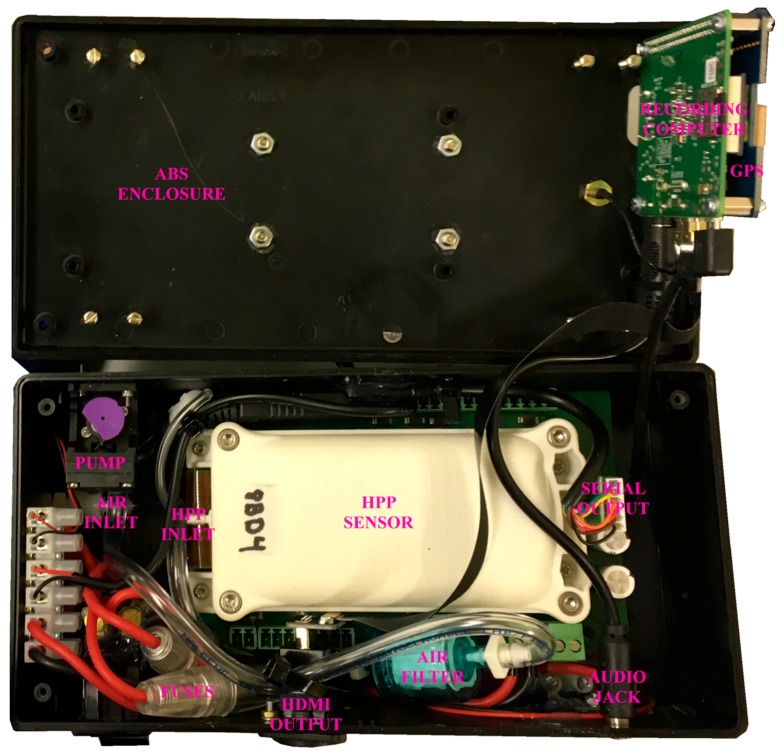
The packaged sensor with the enclosure lid opened and significant operating components annotated.

In order to prepare the HPP methane sensor for UAV sampling, it was first packaged inside an enclosure. The original (unpackaged) SenseAir AB HPP sensor has a mass of 0.327 kg and operates at 12 V. It has a nominal power consumption of 4.2 W and a peak power consumption of 120 W during start-up. Additional power is required at start-up for internal mirror warm-up; the sensor manufacturer recommends a 40 min warm-up period. In this work, the HPP sensor was secured inside an acrylonitrile butadiene styrene (ABS) enclosure (see Figure 1), with a length of 220 mm, width of 110 mm and height of 75 mm (referred to hereafter as the packaged sensor). A tray was secured to the top of the packaged sensor to hold a 12 V detachable lead-acid battery (RS Pro 537-5444, RS Components Ltd., Corby, UK) with a 1.2 Ah capacity. The battery could be secured down using hook and line fastener.

Air was pumped into the HPP sensor using a brushless motor diaphragm pump (NMP 015 M, KNF Neuberger UK Ltd., Oxfordshire, UK). The pump was connected to the external air inlet of the packaged sensor via a barbed 0.03 μm filter (Numatics PTS-B, ASCO Valve, Inc., St. Louis, MO, USA). Polyvinyl chloride (PVC) tubing was used for all internal air connections. The HPP sensor released sampled air into the packaged sensor enclosure, which was vented through several enclosure cavities. The flow rate through the packaged sensor was 1.635 dm^3^ min^−1^ ± 0.002 dm^3^ min^−1^, which was measured using an air flow calibrator (Gilibrator-2 Calibrator, Sensidyne, LP, St. Petersburg, FL, USA).

A recording computer (Raspberry Pi 1 Model A+, Raspberry Pi Foundation, Cambridge, UK) was used to store raw methane mole fraction ([*X*]_0_) measurements from the HPP sensor at 1 Hz, which has a 0.1 ppm resolution. The recording computer was programmed to provide audio messages to the operator, allowing use in the field without a monitor. The computer was connected to the serial port of the HPP sensor via a transistor-transistor logic (TTL) to universal serial bus (USB) serial converter (Future Technology Devices International Ltd., Glasgow, UK). The recording computer was also connected to a USA Global Positioning System (GPS) module (Ultimate GPS HAT for Raspberry Pi, Adafruit Industries, LLC, New York, NY, USA), which was connected to an external antenna. This provided a measurement of GPS time, corresponding to each recorded [*X*]_0_ measurement. In the absence of GPS signal, the GPS module used an internal clock to record time without recording GPS geolocation. If satellite signal were to be lost, GPS geolocation could be derived by interpolating from measurements with good satellite signal, if required. The recording computer logged uncalibrated measurements of [*X*]_0_ and GPS geolocation into a new file as soon as power was supplied to the sensor. Thus measurements made during warm-up were subsequently discarded.

**Figure 2:**
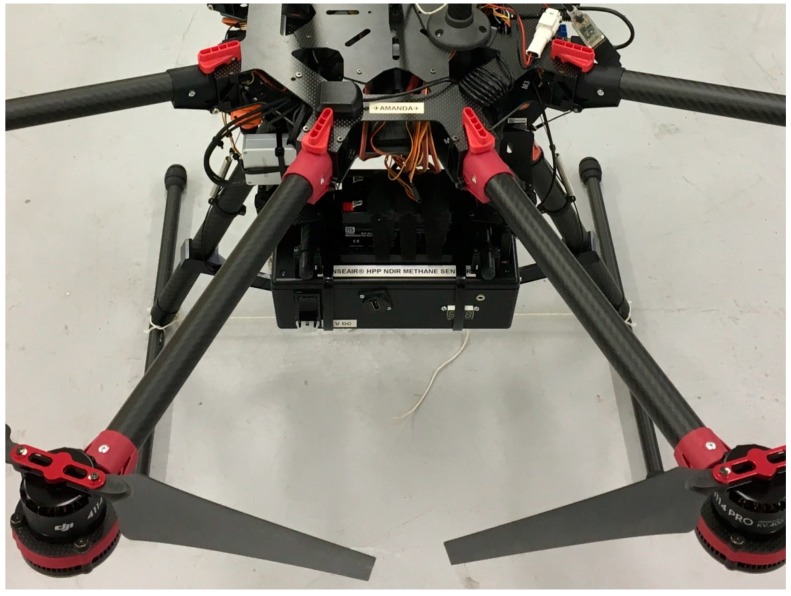
The packaged sensor mounted underneath the centre frame of a DJI Spreading Wings S1000+ octocopter unmanned aerial vehicle.

The packaged sensor had an overall mass of 0.996 kg, excluding a detachable lead-acid (0.512 kg) battery or any power cables. It operated at 12 V (direct current) and was fused at 10 A (above the peak current draw). The packaged sensor was mounted underneath the centre frame of a DJI Spreading Wings S1000+ octocopter UAV (see Figure 2). The sensor was connected to a PVC air inlet. The air entering the air inlet should be as representative as possible of the air being sampled at that point, i.e., air should not be perturbed due to the influence of downwash through the propellers of the UAV. Therefore the air inlet was mounted 0.31 m above the plane of the propellers, where such influence of the UAV is minimal (see Zhou et al. [47] for example). A 4 s lag time was recorded between the UAV air inlet and the HPP sensor measurement cell, which was subsequently corrected for, by correcting the measurement time to be equivalent to sampling time. The packaged sensor payload and UAV had a total take-off mass of 9.2 kg, including the detachable sensor battery and a 16,000 mAh battery for the UAV.

### 2.2. Sensor Characterisation

Before the packaged sensor was used for UAV sampling, its performance was tested in the laboratory. In principle, methane mole fraction can be derived from raw [*X*]_0_ measurements using Equation (1), where *M* is the instrumental gain factor. *M* determines how reported raw methane mole fraction measurements ([*X*]_0_) scale linearly with true methane mole fraction ([*X*]), whereas the offset is an instrumental baseline (lowest) recorded measurement, against which all [*X*]_0_ measurements use as a zero reference.

[*X*] = (*M* ∙ [*X*]_0_) − *offset*. (1)

Although it is possible to derive [*X*] from the packaged sensor, when calculating an emission flux from a facility source, the methane mole fraction enhancement ([*E*]) above a background methane mole fraction ([*X*]_b_) is required. [*X*]_b_ can be acquired from a subset of [*X*]_0_ measurements (see the supplement to Shah et al. [33] for example). [*E*] is then calculated using Equation (2).

[*E*] = *M* ∙ ([*X*]_0_ − [*X*]_b_)). (2)

A key benefit to the use of Equation (2) is that it includes no absolute offset, as this cancels out, requiring calibration for gain factor only. However, Equation (2) implicitly requires that any potential drift in the offset must be small over the duration of sampling relative to the magnitude of any measured mole fraction enhancement. If drifts in the offset are significant compared with any enhancement (for example, greater than 10% of any enhancement itself), any measurements become less meaningful and may result in significant uncertainty in any subsequent flux analysis.

During calibrations to derive *M*, it was important to take into account the effects of variation in water vapour mole fraction in the measurement cell. Such variations are known to have a significant impact on methane mole fraction measurements in infrared sensors, due to spectral overlap of the water vapour continuum in the infrared absorption window of methane, as well as simple dilution effects (see Rella et al. [48] for further discussion of these effects). Equation (2) relies on the assumption that *M* is independent of water vapour mole fraction ([H_2_O]). To test this assumption, *M* was derived at four different [H_2_O] values. *M* was calculated by sampling a low methane mole fraction ([*X*]*_l_*, 1.942 ppm ± 0.001 ppm) and a high methane mole fraction ([*X*]*_h_*, 4.568 ppm ± 0.004 ppm) standard. The standards were produced by blending gas from two cylinders: one contained compressed dry air (Air Products and Chemicals, Inc.) and the other contained approximately 100 ppm of methane (BOC Limited). [*X*]*_l_* and [*X*]*_h_* were measured to high precision using a Los Gatos Research, Inc. Ultra-portable Greenhouse Gas Analyzer (UGGA), which was previously calibrated to traceable standards on the World Meteorological Organisation greenhouse gas scale (see Pitt et al. [49] for a further discussion on the UGGA calibration procedures).

The two gas blends were sampled by the packaged sensor intermittently every 15 min, over a period of at least 4 h. This procedure was first executed using dry gas. It was then repeated by humidifying the gas blends from the cylinders to three fixed dew points, using a dew point generator (LI-610, LI-COR, Inc.). Ten minutes of stable [*X*]_0_ sampling from each 15-min sampling period was used to derive an average low [*X*]_0_ measurement ([*X*]_0*l*_) and an average high [*X*]_0_ measurement ([*X*]_0*h*_) every 30 min. [*X*]_0*l*_ and [*X*]_0*h*_ averages were then interpolated (using a shape-preserving piecewise cubic interpolation) to every 15 min. Thus every [*X*]_0*l*_ measurement had a corresponding [*X*]_0*h*_ measurement and vice-versa. This resulted in 16 pairs of [*X*]_0*l*_ and [*X*]_0*h*_ values for each humidity setting, from which individual gain factors could be derived, using Equation (3).

(3) $$gain\;factor\;=\;\frac{{\left[X\right]}_{h}{\;-\;[X]}_{l}}{{\left[X\right]}_{0h}{\;-\;[X]}_{0l}}.$$

**Figure 3:**
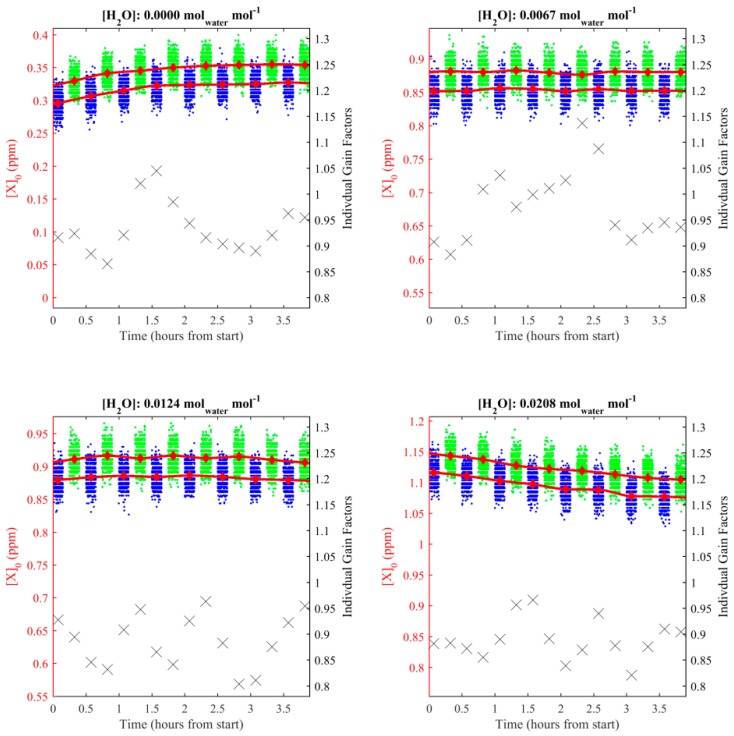
[*X*]_0_ measurements recorded during [*X*]*_h_* sampling (blue dots) and [*X*]*_l_* sampling (green dots) for each humidity setting, plotted against the left-hand axis. Unstable measurements after each gas transition were discarded and are not shown. Averaged [*X*]_0*l*_ and [*X*]_0*h*_ values are plotted as red diamonds against the left-hand axis. [*X*]_0*l*_ and [*X*]_0*h*_ interpolation curves are plotted as red lines. Individual calculated gain factors (black crosses) corresponding to each [*X*]_0*l*_ and [*X*]_0*h*_ pair are plotted against the right-hand axis.

Individual gain factors for each humidity setting are plotted in Figure 3.

**Figure 4:**
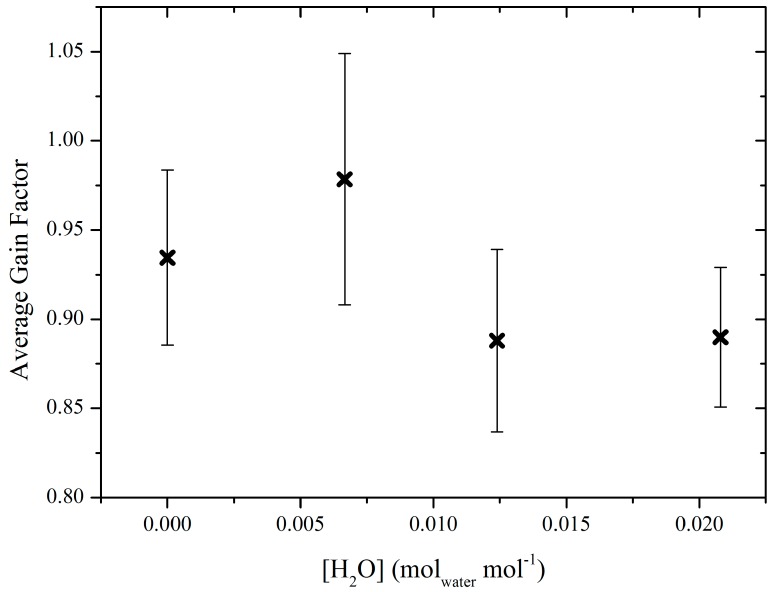
*M* as a function of [H_2_O] (crosses) and associated uncertainties (shown as error bars), representing one standard deviation.

The relative consistency of *M*, compared with the larger changes in [*X*]_0_, shown in Figure 3, suggests that variation in [*X*]_0_ over time is dominated by drifts in the instrumental offset over time, though shorter term drifts in *M* may also pose an issue, especially if the measured mole fraction enhancement is similar to the baseline. As the data in Figure 3 was produced from 15-min data averages, small short-term drifts in *M* cannot easily be identified from this data. The average gain factor at each humidity setting was calculated, along with the standard deviation uncertainty range (see Figure 4). The values in Figure 4 show no obvious linear variation in gain factor up to an absolute humidity of 0.02 mol_water_ mol^−1^, within the bounds of uncertainty. Therefore, *M* was calculated from the average of all 64 individual gain factors at all humidity settings and the uncertainty in *M* (*σ_M_*) was taken to be its corresponding standard deviation 0.92 ± 0.07. While there may be some evidence for a correlation of *M* with humidity, which may become apparent with further testing, this correlation is not possible to de-convolve and quantify meaningfully from the limited calibration data shown in Figure 3. However, the low recorded [*X*]_0_ measurements when the cell was dry in Figure 3 (compared to more humid sampling), indicate that the offset was also small when the cell was dry in our experiments (although further testing may be required to confirm that this would consistently be the case for other NDIR sensors). Thus, the offset may vary both naturally (instrumental drift) and as a function of water mole fraction. As the offset is not included in Equation (2) (used to calculate gain factors), the effect of variation in offset can be ignored when calculating [*E*], provided that one assumes the correlation between offset and water mole fraction to be small. Thus the packaged sensor may be used in flux quantification regardless of the large changes in [*X*]_0_ (at constant [*X*]), provided that one assumes that the change in offset remains small for the duration of sampling, that short-term drifts in *M* remain small and that changes in water mole fraction remain small. If water mole fraction is expected to change significantly during sampling, it may be necessary to quantify its effect on offset and *M* more comprehensively, especially in environments where any methane mole fraction enhancement may be small compared with the magnitude of the offsets seen here. To accurately account for this effect, it may also be necessary to accurately measure absolute humidity during sampling, using a separate humidity sensor.

**Figure 5:**
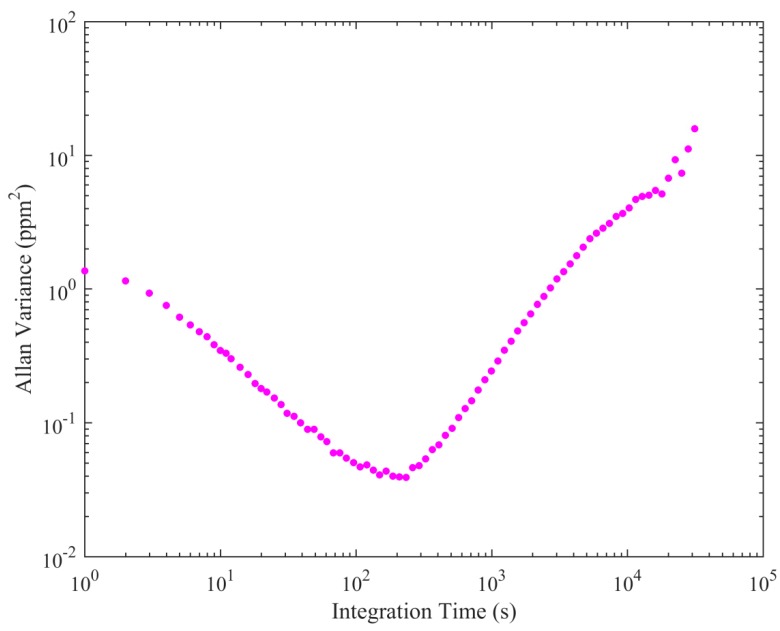
The Allan variance (magenta dots) plotted against integration time on a logarithmic scale.

Next, an Allan variance test was performed to assess the repeatability of measurements and to quantify instrumental precision. Gas from a cylinder of compressed dry air was sampled for 19 h. [*X*]_0_ measurements were converted to [*X*] for this test, using Equation (1) (using an arbitrary offset). The Allan variance results are given in Figure 5, as a function of integration time. This plot shows that the packaged sensor has an optimum integration time of approximately 4 min, above which the effects of instrumental drift begin to dominate over instrumental noise. This 4-min integration time justifies the use of 15-min intervals to calculate *M*, as intervals should be greater than the optimum integration time, from which a suitable uncertainty in *M* can be estimated, based on natural long-term instrumental drift in *M*. Therefore, *σ_M_* can be used to quantify the contribution of long-term (greater than 15 min) instrumental drift towards the nominal uncertainty in [*E*] (Δ[*E*]). The Allan variance test also revealed the Allan deviation precision at 1 Hz (*σ_AV_*) to be ±1.16 ppm. *σ_AV_* can be used to quantify instrumental noise. *σ_AV_* can be combined with *σ_M_* to calculate Δ[*E*] at 1 Hz, using Equation (4).

Δ[*E*] = (*σ_AV_*^2^ + ([*E*] ∙ *σ_M_*)^2^)^0.5^. (4)

Equation (4) assumes no uncertainty in [*X*]*_b_*.

Finally, the e-folding time was calculated; it must be high enough to justify the sampling frequency. The e-folding time represents the time taken for the 63.2% of the contents of the optical cell to be replaced by fresh sampling gas. If it is too large, sampling would be skewed over time. The e-folding time was derived by repeatedly alternating the gas entering the packaged sensor between compressed dry air and air with a high methane mole fraction (~100 ppm). An exponential decay function was fitted to transitions in [*X*]_0_, yielding an e-folding time of 0.7 s ± 0.1 s.

### 2.3. Unmanned Aerial Vehicle Testing

The suitability of the packaged sensor in emission flux quantification was tested by flying the packaged sensor on-board the UAV, on a vertical sampling plane 47 m ± 5 m downwind of a controlled methane release (see the supplement to Shah et al. [50] for details of the controlled release). Nine flight surveys were conducted in August and September 2018 in a field in Little Plumpton, Lancashire, United Kingdom (53.7883° N, −2.9455° E), during which methane was released at an uninterrupted emission rate (see Table 1 for flight survey details). Each flight survey was composed of two individual UAV flights: one to the right of the emission source (projected onto a plane perpendicular to mean wind direction) and one to the left. The UAV sampled on a plane roughly perpendicular to mean wind direction, using pre-programmed waypoints, with a maximum speed of 2 m s^−1^. For cross comparison, an ABB-Los Gatos Research, Inc. Micro-portable Greenhouse Gas Analyzer (MGGA), measuring methane to a high-precision (±2.71 ppb at 10 Hz), was connected to the same UAV using a 150 m tethered air inlet (see Shah et al. [50] for details of this instrument). A stationary on-site sonic anemometer (WS500-UMB Smart Weather Sensor, G. Lufft Mess- und Regeltechnik GmbH) provided live wind vector measurements. The offset angle between the plane perpendicular to mean wind direction and the sampling plane is given in Table 1. The average wind speed recorded by the anemometer during sampling was no higher than 8 m s^−1^, both because the UAV cannot safely fly in high winds and because high winds can more rapidly dilute the emissions at source, thus reducing the magnitude of mole fraction enhancements. The detachable battery was used for uninterrupted power for the full duration of each flight survey, following a 5-min warm-up period on the ground. Prior to this period, the packaged sensor was run for at least one hour to warm-up on the ground, using a large lead-acid battery. The transition between each battery lasted no longer than 30 s, to minimise cooling of the sensor components. The UAV [*X*] sampling results for each flight survey are presented in the next section.

## 3. Results and Discussion

**Figure 6:**
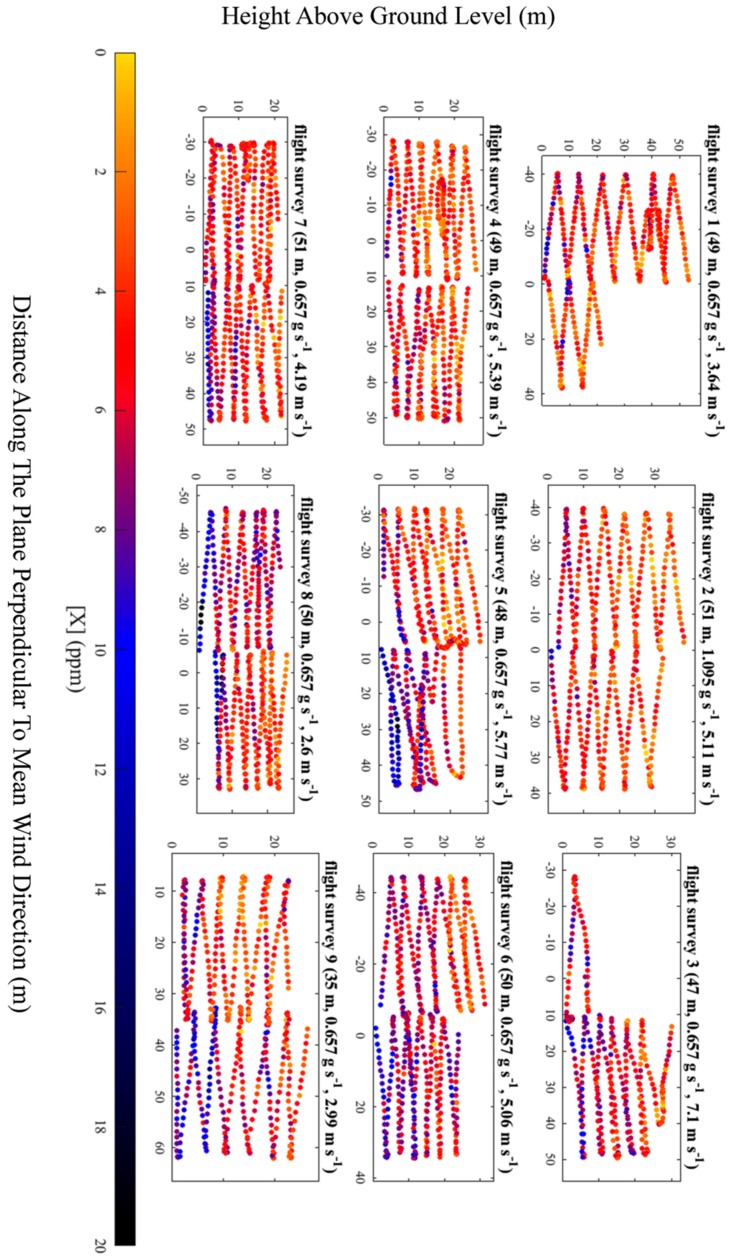
UAV flight tracks for each flight survey. The colour of each dot represents the magnitude of [*X*]. The parallel distance of the sampling plane from the emission source, the true emission flux and the average wind speed at 3.3 m (the height of the anemometer) are given in brackets. In order to calculate [*X*] the lowest [*X*]_0_ measurement from flight survey was treated as the offset (see text).

Geospatially mapped [*X*] measurements acquired during each UAV flight survey were plotted on a plane perpendicular to mean wind direction, to test their suitability in flux quantification (see Figure 6). There was no loss of satellite signal for the duration of sampling. The distance along the plane perpendicular to mean wind direction (*x*) is plotted on the vertical axis and height above ground level (*z*) is plotted on the horizontal axis. Satellite-derived altitude was used to calculate *z*, taking into account the height of the UAV air inlet above the base of the UAV. *x* was derived by converting longitude and latitude into metres and then projecting metric longitude and metric latitude onto a plane perpendicular to mean wind direction (see Shah et al. [33] for further details on deriving *x*). Mean wind direction was derived from the stationary anemometer 3.30 m ± 0.03 m above ground level. [*X*] was derived using Equation (1), where the offset was taken to be the lowest [*X*]_0_ measurement from each flight survey, multiplied by *M*. In principle, the choice of offset should be arbitrary as it cancels out when calculating [*E*] during flux calculation, provided that offset drift is assumed to be small (relative to the magnitude of measured enhancements), for the duration of sampling.

**Figure 7:**
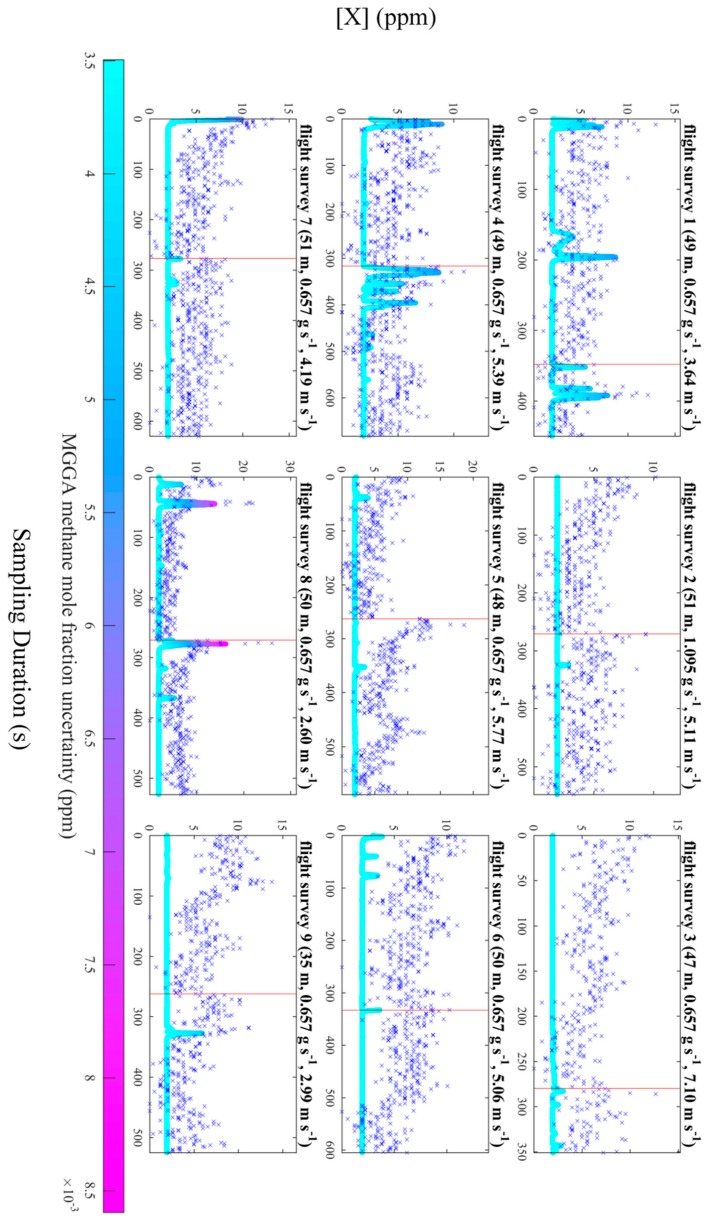
[*X*] for the packaged sensor (blue crosses) and the Micro-portable Greenhouse Gas Analyzer (dots, represented as a solid line) for each flight survey. The colour of each dot represents uncertainty associated with each MGGA measurement. Vertical red lines represent the point at which individual UAV flights are combined.

Flux quantification was attempted using the [*X*] measurements for each survey, presented in Figure 6, using the near-field Gaussian plume inversion (NGI) technique, described by Shah et al. [33]. The NGI method models turbulent plumes sampled near to source, by assuming time-averaged Gaussian turbulent advection of a dynamic time-invariant plume, rather than assuming simple Gaussian downwind dispersion of the time-averaged plume (as in the traditional Gaussian plume model). Meaningful flux quantification using the NGI method failed for each survey as a background mole fraction could not usefully be defined from [*X*] measurements, under the wind speed, due to the combined effects of high levels of instrumental noise (a manifestation of the poor Allan deviation precision) and short-term instrumental drifts which may have occurred over the sampling period of each flight. Flux quantification failed not because of the choice of flux method but rather the capability of the instrument to sample with sufficient resolution above the background. To test this assertion, [*X*] measurements from the packaged sensor were compared to corresponding [*X*] measurements from the MGGA, corrected for lag time (see Figure 7). The data in Figure 7 clearly shows in-plume enhancements above a well-defined background recorded by the MGGA, which could not easily be detected by the packaged sensor. Figure 7 may also indicate the presence of short-term drifts from the packaged sensor, compared to steady a MGGA background.

A reliable (and stable) background is required in Equation (2) to derive measurements of [*E*] from raw [*X*]_0_ measurements. A principle cause of background noise was the high 1 Hz Allan deviation of ±1.16 ppm, in comparison to detectable mole fraction enhancements, at a flux rate of the order of 1 g s^−1^ or 0.1 m^3^ min^−1^. The near-field Gaussian plume inversion technique (alongside most in situ flux quantification techniques) requires there to be clear in-plume mole fraction enhancements over a constant, well-defined background.

While instrumental noise and possible short-term drifts were beyond our control, the magnitude of mole fraction enhancements over the background could be increased by sampling closer to the source. The surveys presented in Figure 6 were conducted on a sampling plane 47 m ± 5 m downwind of the source. Although it was physically possible to sample closer than this, sampling too close to the source can result in the Gaussian plume time-averaged morphology (required in the NGI method) failing to manifest itself well under the timescales of UAV sampling. Thus we conclude that sampling took place as close to the source as possible, in order to maximise background enhancements. However greater mole fraction enhancements may instead be achieved by flying downwind of a source with a higher emission flux or flying under lower wind speeds to reduce dilution of emissions advecting though the sampling plane.

Based on the results in Figure 6, we conclude that the Allan deviation precision was likely the largest constraint on sampling quality, as gradual changes in [*X*] over time were indiscernible from the higher frequency noise, though short-term drifts may have exacerbated the issue of identifying a background. It is entirely possible that reduced noise would still result in difficulty in identifying a background due to short-term drifts, should they be identified. Other factors such as flow of air into the measurement cell were not an issue, as the e-folding time of 0.7 s ± 0.1 s was smaller than the 1 Hz sampling frequency of the packaged sensor. Furthermore, there was no obvious impact on gain factor with changes in water vapour mole fraction, based on the limited calibration data, although a subtle correlation may become evident with further testing. The above discussion ultimately relates to a simple consideration of signal-to-noise; if measured enhancements are sufficiently large compared to the sources of error quantified in this work, the mole fraction measurement can be meaningful and useful for flux calculation by whatever method. To summarise, although the packaged sensor was unsuitable in this test, where only low mole fraction enhancements were measured, it may be used in future to detect larger enhancements from stronger emission plumes, where mole fraction enhancements significantly exceed the Allan deviation precision. As the 1 Hz Allan deviation of the sensor was ±1.16 ppm, we suggest that this sensor may be suitable for sampling mole faction enhancements of the order of at least 10 ppm, (i.e., an order of magnitude greater than the Allan deviation), provided this is large enough to conceal potential short-term drifts.

## 4. Conclusions

A High Performance Platform (HPP) NDIR methane sensor, manufactured by SenseAir AB, was packaged in an enclosure with a total mass of 1.0 kg. The packaged sensor contained a pump, a filter, a GPS module and a recording computer, storing measurements of methane mole fraction and satellite geolocation at 1 Hz. The flow rate through the packaged sensor was 1.635 dm^3^ min^−1^ ± 0.002 dm^3^ min^−1^. The packaged sensor was mounted beneath the centre frame of a small UAV platform with a total take-off mass of 9.2 kg. The lag time between the UAV air inlet and the measurement cell was 4 s.

The packaged senor was calibrated and characterised prior to sampling. It had an e-folding time of 0.7 s ± 0.1 s and a gain factor of 0.92 ± 0.07. The gain factor showed no significant variation in response to changes in water mole fraction of up to 0.02 mol_water_ mol^−1^. The sensor had a 1 Hz Allan deviation precision of ±1.16 ppm, resulting in spurious fluctuations (relative to the higher precision sensor) in measured background methane mole fraction.

The packaged sensor was tested for its suitability in flux quantification by flying it on-board a UAV downwind of a controlled methane release, in nine flight surveys: each survey consisted of two separate UAV flights. Geospatially mapped mole fraction measurements showed that no suitable background could be derived from sampling, due to relatively high instrumental noise (when compared with mole fraction background enhancements). Short-term drifts may have exacerbated the issue, as mole fraction enhancements above a constant background require instrumental drifts to be small for the sampling duration. We therefore conclude that the packaged sensor cannot be used in flux quantification of emissions of the order of 1 g s^−1^, using near-field sampling (47 m ± 5 m from the source), due to a fundamentally poor signal-to-noise ratio. However, the sensor may have useful potential in flux quantification of emissions of greater flux magnitude, of the order of at least 10 g s^−1^.

This work illustrates the challenge for improved methane detection technology in facility-scale flux quantification. Cheap sensors using simple spectroscopic techniques may be incapable of deriving accurate and reliable measurements of methane mole fraction from a UAV platform. Similarly, accurate sensors using advanced spectroscopic techniques are often too heavy to mount onto a small UAV platform. Nevertheless, any future cheap methane sensor that emerges on the market must be thoroughly tested and characterised, prior to its application in scientific research.

## Figures and Tables

**Table 1:** UAV flight times (in Greenwich Mean Time) for each test with corresponding emission flux magnitudes.

Test	Date	Flight 1 Start Time	Flight 1 End Time	Flight 2 Start Time	Flight 2 End Time	Sampling Plane Offset Angle	True Emission Flux (g s^−1^)
1	21.8.2018	15:32:05	15:37:53	15:43:18	15:45:01	+1.9°	0.657
2	23.8.2018	11:21:55	11:26:26	11:31:56	11:36:34	−2.0°	1.095
3	23.8.2018	13:08:24	13:13:04	13:18:24	13:22:29	−13.9°	0.657
4	3.9.2018	12:55:50	13:01:07	13:07:29	13:13:36	−12.2°	0.657
5	3.9.2018	14:05:35	14:09:58	14:15:52	14:21:19	−10.1°	0.657
6	3.9.2018	15:05:38	15:11:11	15:19:39	15:24:12	−7.8°	0.657
7	3.9.2018	16:10:54	16:15:31	16:26:03	16:32:06	+4.6°	0.657
8	4.9.2018	11:52:39	11:57:10	12:05:03	12:09:31	+5.8°	0.657
9	4.9.2018	12:45:19	12:49:41	12:58:22	13:02:46	−46.6°	0.657
